# Dr. Jenner

**Published:** 1823-03-01

**Authors:** 


### Dr. JENNER.

Since our last publication the illustrious Jenner has paid the final debt of Nature! He has gone to the tomb ripe in years, and fraught with honours of the most gratifying kind—the spontaneous plaudits of every enlightened nation on the earth. The discovery of vaccination will render the name of Jenner immortal, even if the vaccine matter itself, from some change in its nature, or revolution in the human system, should ultimately lose its power of protecting man against the variolous poison. For that the vaccine lymph had and still has a protecting power in most, and a modifying power in almost all instances, the proofs are as clear and strong as of any fact or event that has ever happened, or is daily happening on the globe we inhabit. In those countries where the governments are more arbitrary and the subjects less free than in England, vaccination still bids fair to exterminate its baleful enemy; and if the light of reason and the evidence of truth be unable here to overcome stubborn self-will and blind prejudice, the melancholy consequences must not be suffered to detract from the merits either of the discoverer or the discovery. We cannot but hope, therefore, that the profession will evince some lasting testimony of their gratitude and esteem for their departed brother who, when alive, conferred an honour not only on the faculty of which he was a member, but the nation that gave him birth. We are glad to hear that Dr. Baron is entrusted with the unpublished papers of Dr. Jenner, and that they will not be withheld from the public.

Dr. Jenner, youngest son of the Rev. S. Jenner, was born 17th May, 1749, and at the age of 13 was apprenticed to Messrs. Ludlow of Sudbury, surgeons of some distinction. Here he became hypochondriacal, and acquired a morbid susceptibility which, it is said, he retained through life. He afterwards became house pupil to the celebrated John Hunter, and laboured in the construction of the Hunterian Museum. On leaving London Dr. J. retired to Berkley, his native place, and there commenced private practice, where he soon attained considerable eminence. Having read his memoir on the natural history of the cuckoo to the Royal Society, he was soon afterwards elected a fellow. His enquiry into the nature of the cow-pox, commenccd about the year 1776, and it was introduced into the practice of this metropolis in the year 1796, by Mr. Cline. It was soon afterwards introduced into the army and navy, and the medical officers of the latter service presented Dr. Jennor a gold medal representing Apollo, the god of physic, introducing a seaman recovered from vaccine inoculation to Britannia, who, in return, extends a civic crown, on which is inscribed "Jenner." The motto was—*Alba nautis stella refulsit.* The fame of the discoverer and the substantial benefits of the discovery soon pervaded every civilized nation of the earth, and Dr. Jenner received congratulatory epistles from kings and emperors. Even Napoleon, in the zenith of his anti-British hatred, released whole families of English confined as detenu's in France. The parliamentary reward, and the history of vaccination since that period, are sufficiently known. Dr. Jenner died suddenly of apoplexy, on the 26th of January, in the 74th year of his age —*See the Medico-Chirurgical and Philosophical Magazine, No. 2, for Saturday,* 15*th Feb.* 1823.

